# lncRNA HHIP-AS1/HHIP modulates osteogenic differentiation of BM-MSCs by regulating Hedgehog signaling pathway

**DOI:** 10.18632/aging.204381

**Published:** 2022-11-14

**Authors:** Xin-Hua Yin, Xiao-Yuan Wang, Shi-Chang Liu, Liang Yan, Bao-Rong He, Ding-Jun Hao, Ming Yang, Zhong-Kai Liu

**Affiliations:** 1 Department of Spine Surgery, Hong Hui Hospital, Xi’an Jiaotong University College of Medicine, Xi’an, China; 2 Physical Examination Center, Xi'an International Medical Center Hospital, Xi’an, China

**Keywords:** lncRNA HHIP-AS1, HHIP, BM-MSCs, osteogenic differentiation

## Abstract

Background: lncRNA, a type of non-coding RNA, plays an important role in the osteogenic differentiation of bone marrow-derived mesenchymal stem cells (BM-MSCs). In this study, lncRNA and mRNA microarrays were performed to study the change of gene expression during osteogenic differentiation of BM-MSCs. We focused on Hedgehog interacting protein (HHIP), because HHIP mRNA and lncRNA HHIP-AS1 were gradually down-regulated on days 0, 7, and 14 during osteogenic differentiation. In addition, the gene coding *lncRNA HHIP-AS1* is located on the anti-sense of *Hhip* gene, implying the potential interaction between lncRNA HHIP-AS1 and HHIP mRNA.

Methods: BM-MSCs with over-expressed or silenced lncRNA HHIP-AS1 were constructed to explore the biological role of HHIP-AS1 in osteogenic differentiation. BM-MSCs were lysed to determine the alkaline phosphatase activity. Fluorescence *in situ* hybridization and immunofluorescence were performed to analyze HHIP-AS1, HHIP, RUNX2 and osteocalcin.

Results: Overexpression of lncRNA HHIP-AS1 increased HHIP expression, which suppressed Hedgehog signaling pathway, as indicated by the reduction of SMO, Gli1 and Gli2. The suppression of Hedgehog signal was associated with the inhibited osteogenesis. HHIP knockdown abolished the suppression of osteogenesis induced by lncRNA HHIP-AS1 overexpression. Through binding to HHIP mRNA, lncRNA HHIP-AS1 recruited ELAVL1 to HHIP mRNA, whereby increasing the mRNA stability and the protein level.

Conclusions: This study revealed that down-regulation of HHIP due to lncRNA HHIP-AS1 reduction promoted the osteogenic differentiation of BM-MSCs though removing the suppression of Hedgehog signal.

## INTRODUCTION

Mesenchymal stem cells (MSCs) are the most promising cell types in bone regeneration and repair [1, 2]. As an important source of MSCs, bone marrow-derived mesenchymal stem cells (BM-MSCs) are pluripotent. They can differentiate into osteoblasts, chondrocytes and adipocytes [3, 4]. The osteogenic differentiation of BM-MSCs is generally under tightly spatiotemporal controls to maintain skeletal health [5, 6]. However, the impaired osteogenic differentiation can lead to diseases such as osteoporosis. The osteogenic differentiation of BM-MSCs is regulated by many factors, however, its specific mechanism has not been fully elucidated yet.

Long non-coding RNAs (lncRNAs) are a special type of RNA that does not encode proteins or only encodes a small amount of short peptides [7–10]. High-throughput chips and sequencing technologies have identified a large number of lncRNAs from the human genome. Numerous studies have shown that lncRNA has a strong regulatory role in a variety of biological pathways [11, 12]. lncRNA has been reported as an important regulator of gene expression as lncRNAs interact with DNA, RNA and proteins [12]. At present, some studies have shown that lncRNA participates in the regulation of BM-MSCs differentiation [13, 14], but its molecular mechanism has not been extensively investigated. Previous study has revealed that lncRNA Hedgehog interacting protein-antisense (HHIP-AS1) is significantly down-regulated during osteogenic differentiation of BM-MSCs [2]. Interestingly, the gene encoding *lncRNA HHIP-AS1* is located on the anti-sense of *Hhip* gene, indicating the interaction between lncRNA HHIP-AS1 and HHIP mRNA through complementary base pairing. It has been found that lncRNAs can impact the mRNA stabilization when lncRNAs complementary binding to mRNAs [15]. Therefore, we speculated that the interaction between lncRNA HHIP-AS1 and HHIP mRNA also affect HHIP mRNA stability, degradation and expression level of HHIP mRNA in cells, but further verification is needed.

Hedgehog interacting protein (HHIP) is known as a highly conserved, vertebrate-specific HH signaling inhibitor. HHIP interacts with all three HH family members, including Sonic Hedgehog (SHH), Indian Hedgehog (IHH), and Desert Hedgehog (DHH) in the cell membrane, resulting in the suppression of the HH pathway-specific receptor SMO [16–18]. GLI is a transcriptional factor activated by SMO, and mediates biological functions of the HH signaling pathway [19]. Since the HH signaling pathway is closely related to osteogenic differentiation [19, 20], HHIP may also participates in the regulation of osteogenic differentiation by inhibiting HH signal.

As indicated by lncRNA and mRNA microarrays, both lncRNA HHIP-AS1 and HHIP mRNA were gradually down-regulated on day 0, 7, and 14 during osteogenic differentiation *in vitro*. We hypothesized that the down-regulation of HHIP mRNA was related to lncRNA HHIP-AS1. The down-regulation of HHIP might further affect the osteogenic differentiation by regulating the HH signaling pathway. This study aims to test the hypothesis through *in vitro* studies. Our findings may provide new insights in the essential regulatory role of lncRNA HHIP-AS1 in skeletal health and diseases.

## MATERIALS AND METHODS

### BM-MSCs isolation and cell culture

Human BM-MSCs were isolated from three premenopausal female patients (age:20~35) who received fracture surgeries at Hong Hui Hospital, Xi’an Jiaotong University College of Medicine (Xi’an, China). This study was approved by the Ethics committee and the informed consent was obtained from all subjects. The collected bone marrow samples were heparinized and mixed with phosphate-buffered saline (PBS) (GE Healthcare, Madison, WI, USA). After overlaying on Ficoll solution (1.077 g/mL), the re-suspended cells were centrifugated at 1,000 g for 30 min at room temperature to isolate mononuclear cells. BM-MSCs were cultured in α-minimal essential medium (α-MEM) supplemented with 15% fetal bovine serum (FBS) and 1% penicillin/streptomycin (Invitrogen, Carlsbad, CA, USA) in a 5% CO_2_ incubator at 37° C. Culture the cells in an incubator containing 5% CO_2_ and 95% air at 37° C. After 48 h, the medium was changed to remove non-adherent cells. An additional 4 day-culture was performed by changing the medium until BM-MSCs were confluent. The cells were digested with 0.25% trypsin-EDTA (Invitrogen) and reseeded in 6-well plates (5 × 10^5^ cells each well) or 12-well plates (1 × 10^5^ cells each well).

### Plasmid construction and transfection

When the cells were seeded at 80% confluence, the third-passage BM-MSCs were transfected. siRNAs targeting HHIP-AS1 (si-HHIP-AS1), HHIP (si-HHIP), ELAVL1 (si-ELAVL1) or a control non-targeting siRNA (NC) (Santa Cruz, Tokyo, Japan) were transfected into BM-MSCs using Lipofectamine RNAi MAX reagent (Thermo Fisher Scientific, Waltham, MA, USA) as per the manufacturer’s procedure. To induce the overexpression of HHIP-AS1, the oligonucleotides of full-length HHIP-AS1 (HHIP-AS1), overlapping sequence between HHIP-AS1 and HHIP (HHIP-AS1 OL), and non-overlapping sequence between HHIP-AS1 and HHIP (HHIP-AS1 non-OL) from GenePharma (Shanghai, China) were transfected into BM-MSCs using Lipofectamine RNAi MAX reagent.

### Osteogenic differentiation of BM-MSCs

For osteogenesis, the third-passage BM-MSCs were resuspended in α-MEM containing 15% FBS, 1% penicillin/streptomycin, 50 μg/mL ascorbic acid, 10 mmol/L β-glycerophosphate, and 0.1 mM dexamethasone. The cells were plated in 6-well plates at a density of 5 × 10^5^ cells per well. Cells were cultured at 37° C in a 5% CO_2_ incubator for 2 weeks.

After culturing in osteogenic medium for 14 days, BM-MSCs were tested for alkaline phosphatase (ALP) activity and mineralization. To stain ALP, the cells were fixed in 4% polyoxymethylene for 15 min and incubated with 0.1 M Tris buffer (pH 9.3) containing 0.25% naphthol AS-BI phosphate (Sigma-Aldrich, St Louis, MO, USA) and 0.75% Fast Blue BB (Sigma-Aldrich). The ALP activity was quantified using a commercial kit following the manufacturer’s protocol (Cell Biolab, San Diego, CA, USA). The optical density was measured at 450 nm with a spectrophotometer (Thermo Fisher Scientific).

For alizarin red staining, cells were fixed in 4% polyoxymethylene for 15 min and stained with 1% Alizarin red S (pH 4.2) (Sigma-Aldrich) for 5 min. The mineralized matrix stained with alizarin red were rinsed repeatedly with 10% cetylpyridinium chloride in 10 mM sodium phosphate (pH 7.0). With different calcium dilutions, a standard calcium curve was firstly developed using a spectrophotometer (Thermo Fisher Scientific) at 562 nm, and the calcium concentration in cells was then determined [21, 22]. The final calcium level in each group was normalized to the total protein concentration detected in the duplicate plate.

### Microarray and bioinformatics analysis

The intersections of the element lists of BM-MSCs collected at day 0, 7 and 14 during the osteogenetic induction were calculated using the online tool BIOINFORMATICS and EVOLUTIONARY GENOMICS (http://bioinformatics.psb.ugent.be/webtools/Venn/). IntaRNA algorithm was used to predict the targeting site between lncRNA HHIP-AS1 and HHIP mRNA [23]. Gene ontology analysis was performed using Blast2GO (https://www.blast2go.com/) [24] to clarify the biological function of the differentially expressed lncRNAs. Meanwhile, the number of differential proteins was counted at the level of GO secondary function annotation. Through Fisher’s exact test (*p* < 0.05), GO functional enrichment analysis was performed to reveal the overall functional enrichment characteristics of all differentially expressed proteins and its significance level. Finally, the target GO entries were obtained. At day 7 or 14 of osteogenetic induction, the differentially expressed lncRNAs were identified when their down-regulated fold-change was less than 0.5 or the up-regulated fold-change was greater than 2 (*p*-value < 0.05).

### Reverse transcription-quantitative PCR (RT-qPCR)

Total RNA from the cells was extracted using Trizol (Invitrogen) according to the manual. RNA concentration was measured using a NanoDrop 2000 (Thermo Fisher Scientific). cDNA was prepared using a PrimeScript RT reagent Kit (Takara, Tokyo, Japan) with gDNA eraser (Takara). The RT-qPCR to quantify HHIP-AS1 or HHIP was performed using SYBR Premix Ex Taq II (Takara) on the CFX96 real-time system (Bio-Rad, Hercules, CA, USA). The relative gene expression was normalized to β-actin using the 2^-ΔΔCt^ method. The specific primers used in this study include: HHIP Forward 5’-TACACTTGCCGAGGCCATATT-3’ and Reverse 5’-CCCACTCACAACCTCCTGAAT-3’; HHIP-AS1 Forward 5’-TCTTCTGCTCACACCACCAC-3’ and Reverse 5’-TGCCAGCTCATACAAGATGC-3’; β-actin Forward 5’-CATGTACGTTGCTATCCAGGC-3’ and Reverse 5’-CTCCTTAATGTCACGCACGAT-3’.

### Western blot

After 0-21 days of induction, cells were collected, rinsed in PBS, and then lysed in RIPA buffer (Pierce, Rockford, IL, USA) containing protease and phosphatase inhibitors cocktail on ice. Proteins in cell membrane and cytoplasm were extracted using Minute™ Plasma Membrane Protein Isolation and Cell Fractionation Kit (Beijing, China). The supernatants were collected and the protein content was determined using the Bradford Protein Assay Kit (Bio-Rad). A total of 30 μg protein was loaded onto the 10% sodium dodecyl sulfate-polyacrylamide gel electrophoresis (SDS-PAGE). The isolated proteins were electro-transferred onto PVDF membranes (Millipore, Billerica, MA, USA). The membrane was sealed with 5% skimmed milk and then incubated with rabbit-derived antibodies against HHIP, RUNX2, osteocalcin (OCN), ALP, osteopontin (OPN), ATP1A1 and β-actin at a 1:1,000 dilution in 5% fetal bovine serum (FBS) overnight at 4° C. All membranes were incubated with horseradish peroxidase-conjugated anti-rabbit secondary antibodies at a dilution of 1:3,000 for 30 min at room temperature. The protein bands were visualized with the Enhanced Chemiluminescence Kit (Millipore). Image J was used to digitize the protein signaling. β-actin was selected as a control for protein loading.

### RNA pull-down assay

Bio (biotinylated)-NC, bio-HHIP-AS1 OL, bio-HHIP-AS1 non-OL labelled with RNA Labeling Mix (Roche, Mannheim, Germany) *in vitro* were transfected into BMSCs. The cells were lysed for 1h and collected using streptavidin-coupled magnetic bead (Invitrogen). The protein-bio/RNA-magnetic bead complex was isolated using high salt elution. The protein-bio/RNA was extracted and purified with TRIzol Kit, followed by qRT-PCR of HHIP mRNA.

### RNA stability assay

To measure the RNA stability, 5 g/ml actinomycin D (Sigma-Aldrich) was added to BM-MSCs to inhibit the transcription. Cells were incubated for 0 h, 5 h, 10 h, and 15 h. At each time point, RNA was harvested and quantified by qRT-PCR as described above. Transcript levels were plotted by an appropriate nonlinear regression curves using a one phase decay equation. RNA decay rate constant (k) was calculated by fitting an exponential curve to the data set (y = a*e-kt; y is the relative amount of RNA, and t is time). The turnover rate of mRNA was estimated according to previous reference [25]. The half-life was then calculated according to the equation t1/2 = ln(2)/k(26). The normalizer transcript 18s rRNA that does not decay over the course was used as control.

### Immunofluorescence assay

The cells were rinsed twice using sterilized PBS, and then fixed in 3.7% formaldehyde for 10 min. The coverslips were rinsed in pre-cold PBS and then permeabilized using 0.5% Triton X-100 at 0° C for 20 min. Cells were incubated with primary antibodies against HHIP, RUNX2 or osteocalcin overnight at 4° C. The goat anti-rabbit IgG second antibody was used to probe the primary antibody for 1 h at room temperature. Cells were rinsed in PBS, counterstained with 4’,6-diamidino-2-phenylindole (DAPI, 1: 1,000), and rinsed in 0.1% Triton X-100. ProLong™ Gold Antifade Mountant (Invitrogen) was used to protect fluorescent dyes from fading. The slides were examined on a DMI4000B confocal microscopy equipped with a camera (Leica, Wetzlar, Germany).

### Fluorescence *in situ* hybridization (FISH) analysis

Probes for lncRNA HHIP-AS1 and U6 were synthesized by Biosearch Technologies (Novato, CA, USA). RNA hybridization was performed at 40° C for 4 hrs, and mixed with 50 mL washing solution containing 20 mmol Tris-HCl (pH 7.2), 900 mmol NaCl and 0.01% SDS. The mixture was incubated at 48° C for 30 min. Then 50 μL of DAPI (1: 1,000) was used to overlay the air-dried filters, and incubated for 5 min. The excessive DAPI stain was removed using 80% ethanol solution. The filters were observed by DMI4000B confocal microscopy.

### RNA-immunoprecipitation (RIP) assay

RIP assay was performed in lncRNA HHIP-AS1-overexpressed and silenced BM-MSCs and the control cells and using Magna RIP RNA-binding protein immunoprecipitation kit (Millipore) according to manufacturer’s instructions. RNA extracted from total cell lysates, and incubated with IgG (as a negative control) and anti-ELAVL1 antibody. The immunoprecipitates were subjected to qPCR assay to determine the enrichments of HHIP-AS1 and HHIP mRNA in the immunoprecipitates.

### Statistical analysis

All data were shown as means ± standard deviation (SD). IBM SPSS Statistics 21.0 (IBM, Armonk, NY, USA) was used to perform statistical analysis with One-Way or Two-Way ANOVA corrected by Dunnett. *P*-values less than 0.05 were considered as statistical significance.

### Availability of data and material

The datasets generated/analyzed in the present study are available upon reasonable request from the corresponding author.

## RESULTS

### lncRNA HHIP-AS1 and HHIP are continuously down-regulated during osteogenic differentiation of BM-MSCs

**Figure 1:**
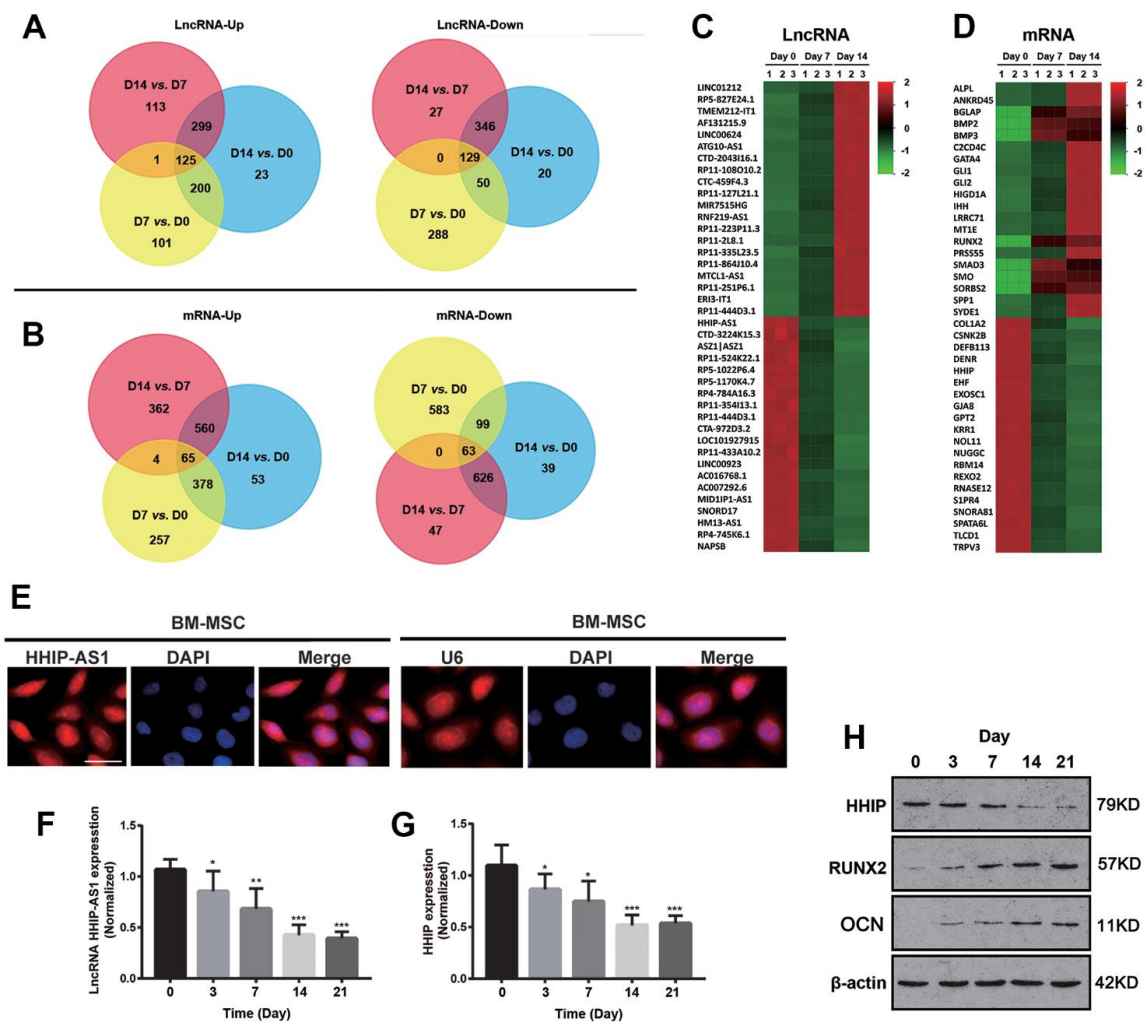
**lncRNA HHIP-AS1 and HHIP are continuously down-regulated during osteogenic differentiation of BM-MSCs.** Up-regulated or down-regulated (**A**) lncRNAs and (**B**) mRNAs were analyzed by Venn diagram during osteogenic differentiation at days 0, 7 and 14 of osteogenic differentiation of BM-MSCs. Heatmap of (**C**) lncRNA and (**D**) mRNA changes at days 0, 7 and 14 of osteogenic differentiation of BM-MSCs. (**E**) FISH showing that lncRNA HHIP-AS1 was expressed in the nucleus and cytoplasm of BM-MSCs at day 7 of osteogenic differentiation of BM-MSCs. mRNA expression level of (**F**) lncRNA HHIP-AS1 and (**G**) HHIP during osteogenic differentiation. (**H**) Protein expression level of HHIP, RUNX2, and osteocalcin during osteogenic differentiation of BM-MSCs. The histogram data for each group are the average of three independent replicates; bars represent standard deviation; *P < 0.05, **P < 0.01, ***P < 0.001; Scale bar = 10 μm.

BM-MSCs were cultured in osteogenic medium and collected on day 0, 7 and 14 during osteogenic differentiation for high-throughput lncRNAs and mRNAs microarrays. A total of 125 lncRNAs were continuously up-regulated, while 129 lncRNAs were down-regulated (Figure 1A). There were 65 and 63 genes were up-regulated and down-regulated, respectively (Figure 1B). The top 20 differentially expressed lncRNAs and mRNAs were marked in heatmaps (Figure 1C, 1D). The top down-regulated lncRNA was HHIP-AS1 during the osteogenic differentiation. Coincidentally, HHIP mRNA was the fifth most significantly down-regulated mRNA during the osteogenic differentiation. Results from FISH assay showed that lncRNA HHIP-AS1 was located in both nucleus and cytoplasm of BM-MSCs (Figure 1E). A U6 probe, in cell nucleus, was used as the positive control. We further examined the expression level of lncRNA HHIP-AS1 and HHIP at different time points (on days 0, 3, 7, 14, and 21) during osteogenic differentiation of BM-MSCs using PCR assay. The results confirmed that both were continuously down-regulated at the mRNA level during osteogenic differentiation (Figure 1F, 1G). As indicated by western blot, HHIP protein level was gradually decreased during osteogenic differentiation. However, protein levels of RUNX2 and osteocalcin, the markers of osteogenic differentiation, were gradually increased (Figure 1H). The change was most obvious on day 7 and 14, thus the following gene intervention experiments were performed on day 7 and 14 after the induction.

### lncRNA HHIP-AS1 up-regulates HHIP and inhibits osteogenic differentiation of BM-MSCs

**Figure 2:**
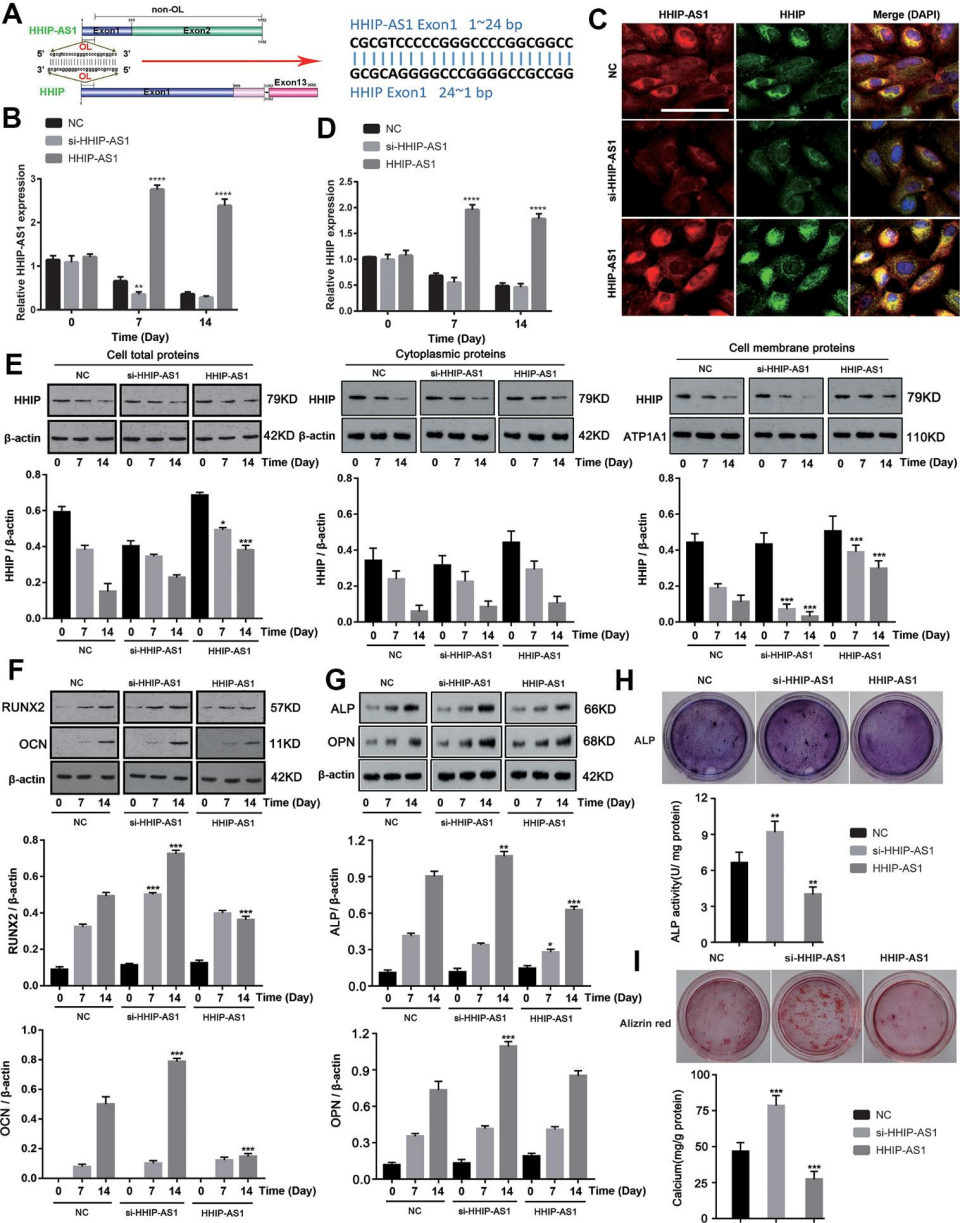
**lncRNA HHIP-AS1 up-regulates HHIP expression and inhibits osteogenic differentiation of BM-MSCs.** (**A**) The complementary sequence of the first exon of HHIP-AS1 and the first exon of HHIP. (**B**) lncRNA HHIP-AS1 in si-HHIP-AS1 or HHIP-AS1 transfected BM-MSCs was detected by PCR. (**C**) Fluorescence images of lncRNA HHIP-AS1 (Red), HHIP protein (Green) and DAPI (Blue) in BM-MSCs transfected with NC, si-HHIP-AS1 and HHIP-AS1. (**D**) HHIP mRNA in si-HHIP-AS1 or HHIP-AS1 transfected BM-MSCs detected by PCR. (**E**) HHIP protein in cell or in cell fractions of cytoplasm and cell membrane was detected by western blot after overexpression or knockdown of lncRNA HHIP-AS1. (**F**) RUNX2 and OCN as well as (**G**) ALP and OPN protein levels in cells were detected by western blot after overexpression or knockdown of lncRNA HHIP-AS1. (**H**) ALP and (**I**) alizarin red staining after 14 days of osteogenic induction of BM-MSCs. The histogram data for each group are the average of three independent replicates; bars represent standard deviation; *P < 0.05, **P < 0.01, ***P < 0.001, ****P < 0.0001. Scale bar = 10 μm. Note: Protein levels in lncRNA HHIP-AS1 knockdown and overexpression groups on day 7 were compared to those in NC groups on day7. Similarly, protein levels in lncRNA HHIP-AS1 knockdown and overexpression groups on day 14 were compared to those in NC groups on day14.

As both HHIP-AS1 and HHIP were significantly down-regulated, we aimed to further explore their roles in osteogenic differentiation of BM-MSCs. We found that the first exon of *HHIP-AS1* gene is completely complementary to the 1-24 bp region of the first exon of *Hhip* gene (Figure 2A). We constructed HHIP-AS1-silenced and HHIP-AS1-overexpressed BM-MSCs (*P* < 0.01, or *P* < 0.0001, Figure 2B). Through FISH and immunofluorescence assays, we observed that lncRNA HHIP-AS1 deficiency was associated with less HHIP protein, while HHIP-AS1 overexpression was observed with more HHIP (Figure 2C). We further performed PCR and western blot to quantify HHIP mRNA and protein levels after lncRNA HHIP-AS1 knockdown and overexpression. lncRNA HHIP-AS1 overexpression increased HHIP mRNA and protein on day 7 and 14 (*P* < 0.05, *P* < 0.001, *P* < 0.0001, Figure 2D, 2E). Interestingly, knockdown of lncRNA HHIP-AS1 had a moderate effect on the HHIP protein in the cells and cytoplasm, but caused a notable reduction of HHIP protein in the cell membrane (*P* < 0.05, *P* < 0.001, Figure 2E). lncRNA HHIP-AS1 overexpression increased HHIP protein in the cells and the cell membrane, but had no effect on HHIP protein in the cytoplasm. After knocking down lncRNA HHIP-AS1 in BM-MSCs, the protein levels of RUNX2 increased on days 7 and 14, while other markers of osteogenic differentiation, including OCN, ALP and OPN were only increased on day 14 (Figure 2F, 2G). BM-MSCs overexpressing lncRNA HHIP-AS1 showed the reduction of RUNX2 and OCN on day 14 and of ALP on days 7 and 14 (*P* < 0.05, *P* < 0.001). HHIP-AS1 overexpression inhibited ALP activity and calcium mineralization in BM-MSCs (*P* < 0.01, *P* < 0.001) while HHIP-AS1 deficiency enhanced ALP activity and calcium mineralization, suggesting that lncRNA HHIP-AS1 overexpression blocks osteogenic differentiation of BM-MSCs (Figure 2H, 2I). In summary, lncRNA HHIP-AS1 overexpression inhibits osteogenic differentiation of BM-MSCs, and the biological role of lncRNA HHIP-AS1 may be associated with HHIP.

### HHIP protein inhibits osteogenic differentiation of BM-MSCs

**Figure 3:**
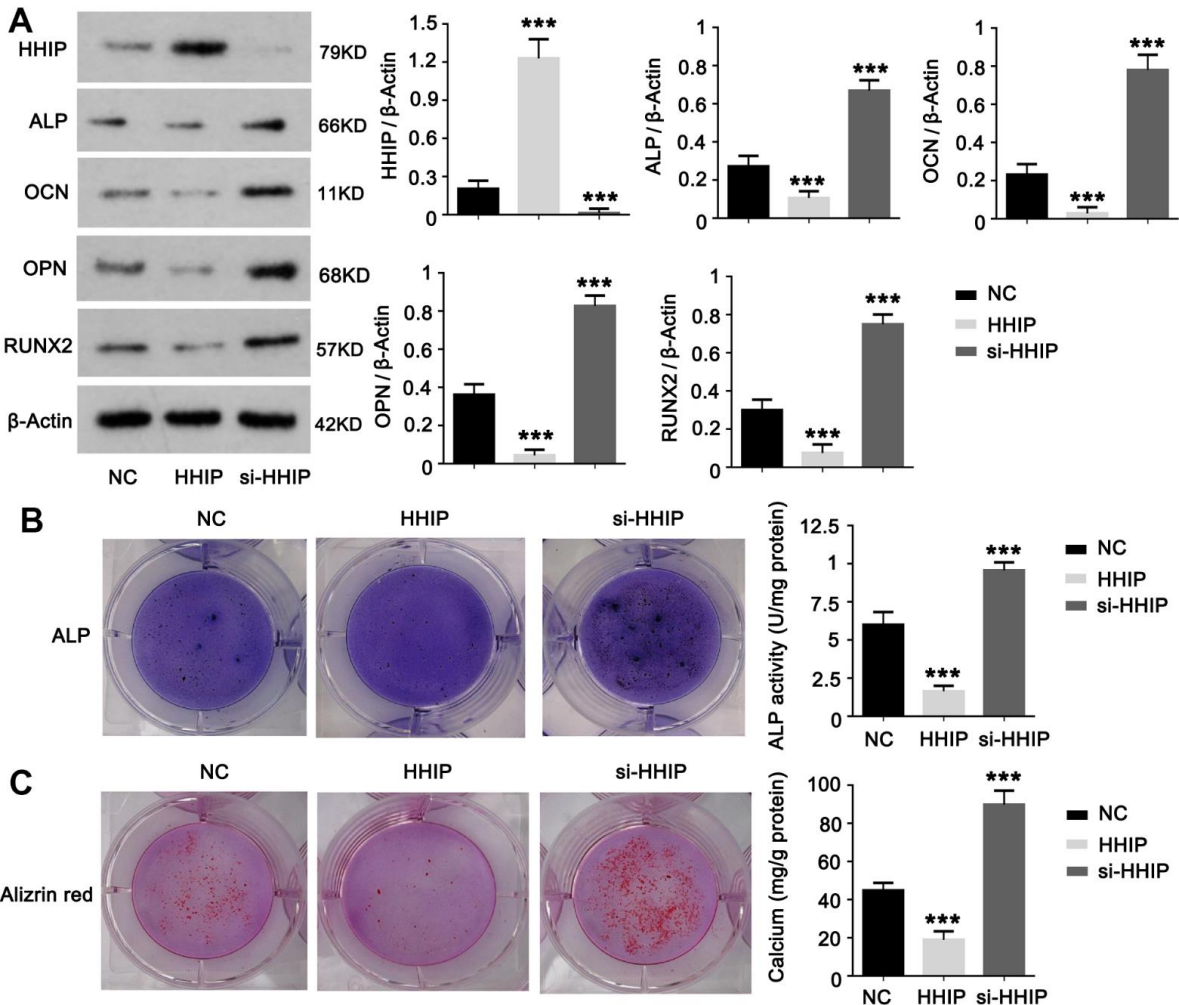
**HHIP protein inhibits osteogenic differentiation of BM-MSCs.** (**A**) We constructed HHIP-silenced (si-HHIP) and HHIP-overexpressed (HHIP) BM-MSCs. RUNX2, OCN, ALP and OPN protein levels were detected after 14 days of osteogenic induction of the BM-MSCs. (**B**) ALP and (**C**) alizarin red staining after 14 days of osteogenic induction of BM-MSCs. *P < 0.05, **P < 0.01, ***P < 0.001.

To understand the role of HHIP in osteogenic differentiation of BM-MSCs, we constructed HHIP-silenced and HHIP -overexpressed BM-MSCs. HHIP overexpression decreased protein levels of RUNX2, OCN, ALP and OPN on day 14, while HHIP knockdown increased the protein levels of RUNX2, OCN, ALP and OPN (*P* < 0.001, Figure 3A). HHIP overexpression inhibited ALP activity and calcium mineralization in BM-MSCs, while HHIP depletion increased ALP activity and calcium mineralization (*P* < 0.001, Figure 3B, 3C).

### lncRNA HHIP-AS1 inhibits osteogenic differentiation of BM-MSCs by increasing HHIP RNA stability

**Figure 4:**
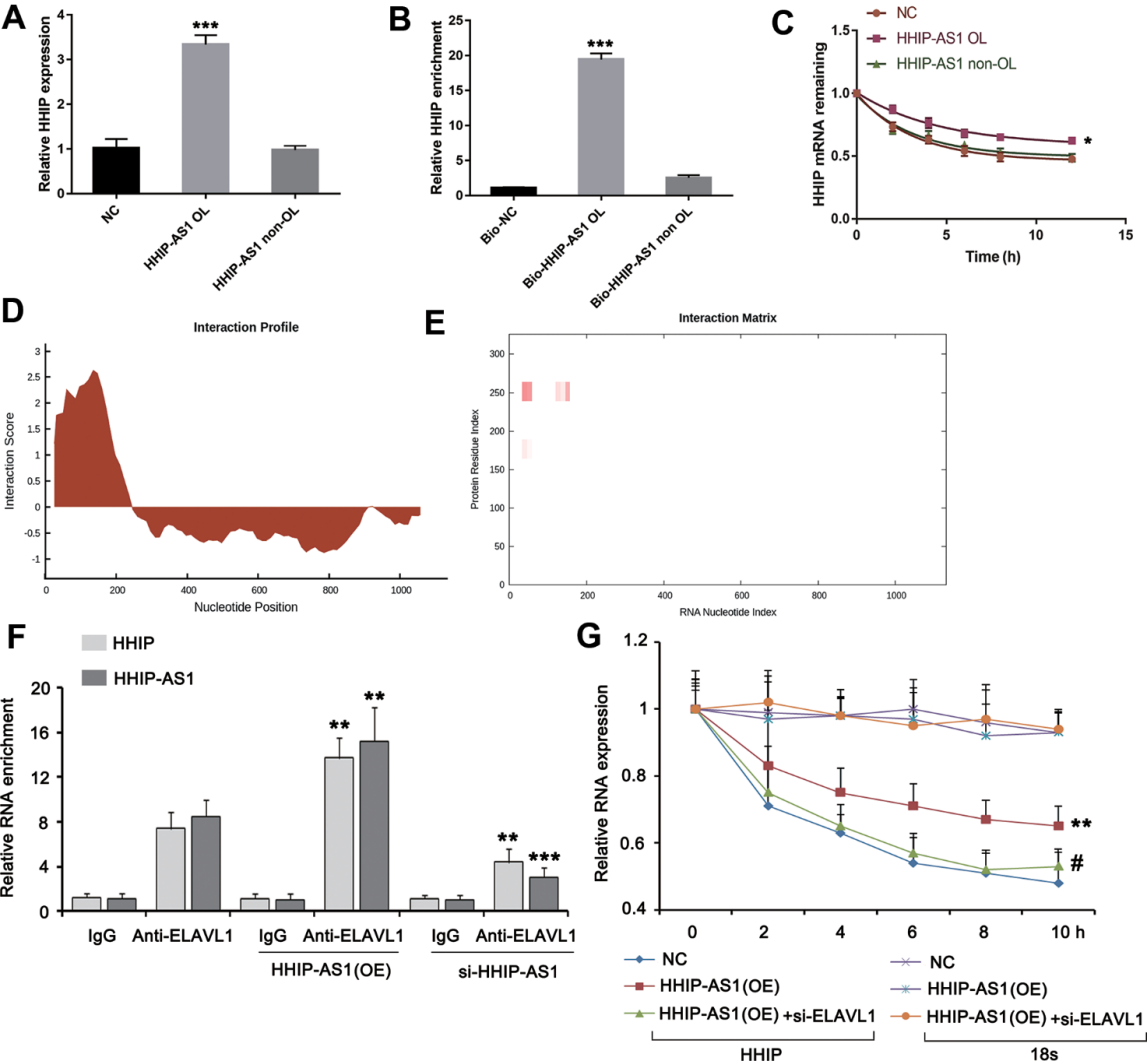
**lncRNA HHIP-AS1 increases HHIP mRNA stability by ELAVL1.** (**A**) BM-MSCs transfected with overlapping sequence or non-overlapping sequence of lncRNA HHIP-AS1 and HHIP mRNA were analyzed by PCR. (**B**) qPCR validation of HHIP enrichment versus input after RNA pull down by two different specific probes (Bio-HHIP-AS1 OL and Bio-HHIP-AS1 non-OL) as compared to a non-specific one (Bio-NC) in BM-MSCs. (**C**) Measurement of the stability of HHIP mRNA by RT-qPCR in the presence of transcriptional inhibitor (actinomycin D) at the indicated time as compared to an internal control 18S rRNA. (**D, E**) Using the web (http://service.tartaglialab.com/page/catrapid_group), we found that ELAVL1 has high affinity to the lncRNA HHIP-AS1, especially with the region that is complementary with HHIP mRNA. (**F**) RIP assay was performed in lncRNA HHIP-AS1-overexpressed and silenced BM-MSCs and the control cells. The qPCR assay was further performed to determine the enrichments of HHIP-AS1 and HHIP mRNA in ELAVL1 protein. IgG is as a negative control. (**G**) BM-MSCs were transfected with lncRNA HHIP-AS1 overexpression vector alone or in combination with ELAVL1 siRNA. Measurement of the stability of HHIP mRNA by RT-qPCR in the presence of transcriptional inhibitor (actinomycin D) at the indicated time as compared to an internal control 18S rRNA. The histogram data for each group are the average of three independent replicates; bars represent standard deviation; *P < 0.05, **P < 0.01 and ***P < 0.001 vs. NC or BM-MSCs without transfection; ^#^*P* < 0.01 vs. BM-MSCs transfected with lncRNA HHIP-AS1 overexpression vector.

It has been reported that lncRNA antisense can form complementary RNA duplex with the target sense gene and increase target gene stability and its expression [15]. As predicted using IntaRNA algorithm, lncRNA HHIP-AS1 shared the complementary sequence with the first exon of HHIP. Then we constructed the recombinant plasmids carrying the overlapping or non-overlapping sequences of lncRNA HHIP-AS1 and HHIP. Results from qRT-PCR analysis showed that HHIP-AS1 OL, but not HHIP-AS1 non-OL, contributed to a significant increase in HHIP transcription (*P* < 0.001) (Figure 4A). The RNA pull-down experiment confirmed that the overlapping regions of lncRNA HHIP-AS1 and HHIP mRNA can be complementarily combined (*P* < 0.001) (Figure 4B). The results of RNA stability experiments showed that the half-life of HHIP was significantly prolonged and the stability was increased after overexpression of the overlap region of HHIP-AS1 (*P* < 0.05) (Figure 4C). These data suggested that lncRNA HHIP-AS1 binds to HHIP mRNA, leading to increased stability of HHIP mRNA. To understand how lncRNA HHIP-AS1 enhances HHIP mRNA stability, we searched the RBP (RNA-binding protein) in lncRNA HHIP-AS1 through the web (http://rbpdb.ccbr.utoronto.ca//index.php). The web showed a few of RBPs binding to lncRNA HHIP-AS1 (shown in Supplementary File 1). Among the RBPs, we focused on ELAVL1 (also termed as HuR), because it has been confirmed to enhance mRNA stability. The interaction between lncRNA HHIP-AS1 and Supplementary File 1 ELAVL1 was further identified by using the web (http://pridb.gdcb.iastate.edu/RPISeq/#) (shown in Supplementary File 1). Using the web (http://service.tartaglialab.com/page/catrapid_group), we found that ELAVL1 has high affinity to the lncRNA HHIP-AS1, especially within the region that is complementary with HHIP mRNA (Figure 4D, 4E and the Supplementary File 1). Through RIP assay, we found that HHIP-AS1 overexpression increased the abundance of HHIP mRNA in ELAVL1 protein, while silencing lncRNA HHIP-AS1 decreased the abundance of HHIP mRNA in ELAVL1 protein (*P* < 0.01) (Figure 4F). Overexpression lncRNA HHIP-AS1 enhanced the HHIP mRNA stability, however silencing ELAVL1 abolished the effect of lncRNA HHIP-AS1 (Figure 4G).

**Figure 5:**
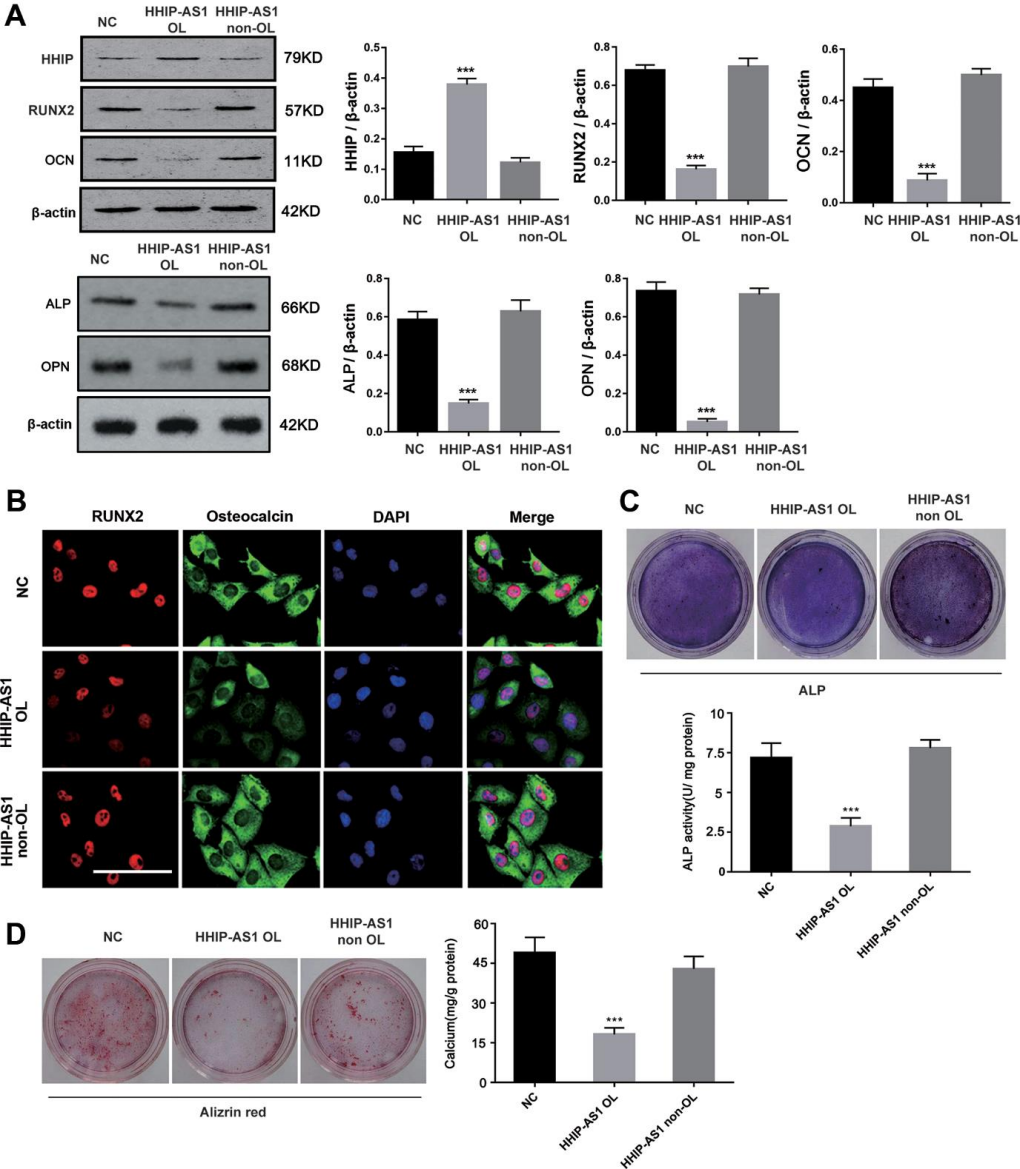
**HHIP-AS1 OL inhibits osteogenic differentiation of BM-MSCs by HHIP.** (**A**) HHIP, RUNX2, OCN, ALP and OPN protein expression level after overexpression lncRNA HHIP-AS1 OL or non OL by western blot. (**B**) Immunofluorescence images of RUNX2 (red), osteocalcin (Green), and DAPI (Blue) in BM-MSCs transfected with NC, HHIP-AS1 OL, or HHIP-AS1 non-OL. (**C**) ALP and (**D**) alizarin red staining after 14 days of osteogenic induction of BM-MSCs. The histogram data for each group are the average of three independent replicates; bars represent standard deviation; *P < 0.05, ***P < 0.001; Scale bar = 10 μm.

Western blot results showed that lncRNA HHIP-AS1 OL increased HHIP protein (*P* < 0.001), but decreased RUNX2, OCN, ALP and OPN proteins (*P* < 0.001) (Figure 5A). Immunofluorescence results showed that lncRNA HHIP-AS1 OL instead of HHIP-AS1 non-OL decreased the accumulation of RUNX and osteocalcin in both nucleus and cytoplasm (Figure 5B). ALP and alizarin red staining results indicated that the osteoblastic differentiation of BMSCs was suppressed after overexpression of lncRNA HHIP-AS1 OL, but was not lncRNA HHIP-AS1 non-OL (Figure 5C, 5D). These results indicated that lncRNA HHIP-AS1 inhibits osteogenic differentiation of BM-MSCs by increasing HHIP RNA stability through recruiting ELAVL1.

### lncRNA HHIP-AS1 inhibits osteogenic differentiation of BM-MSCs by inhibiting Hedgehog signaling pathway

**Figure 6:**
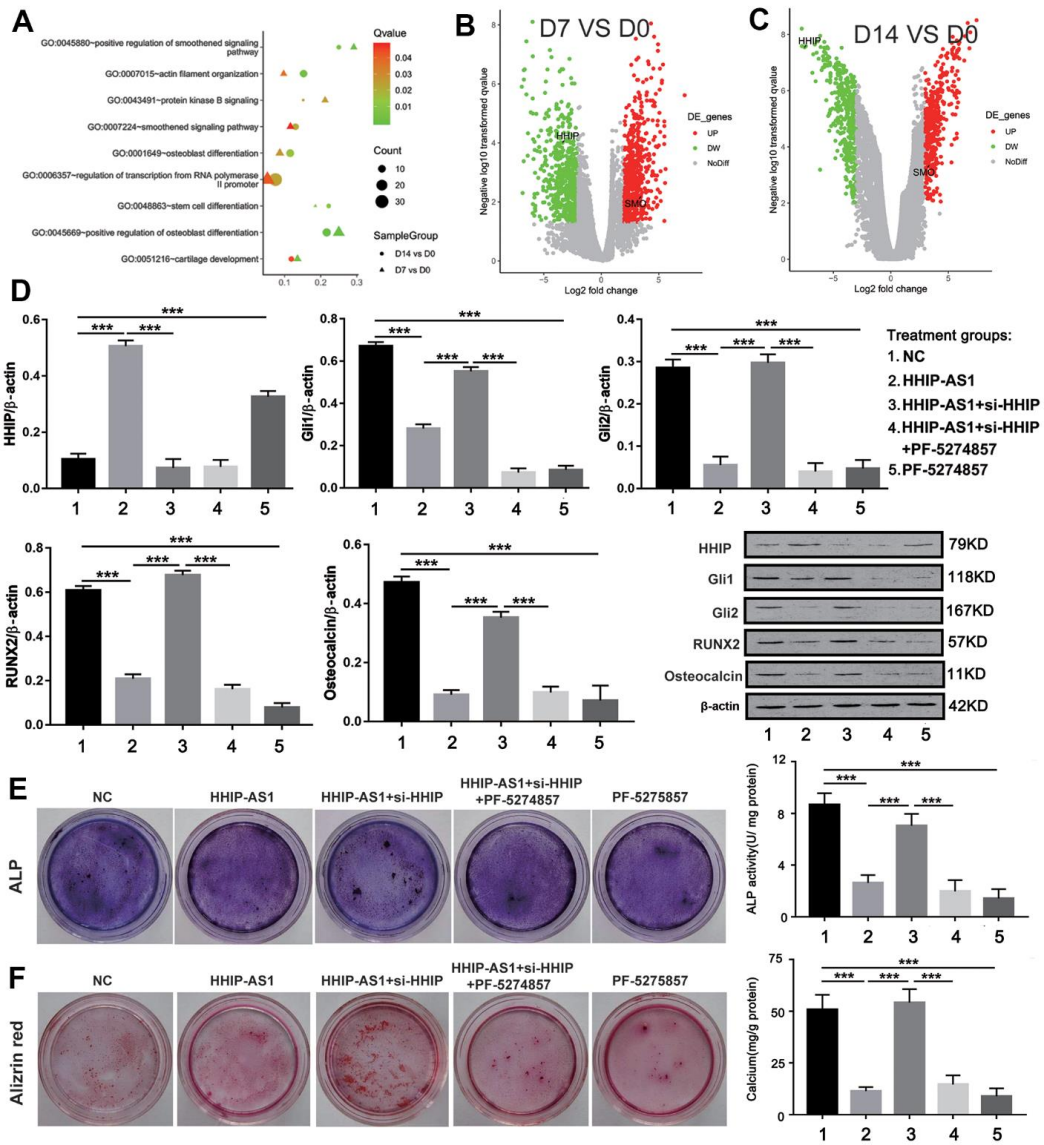
**lncRNA HHIP-AS1 inhibits osteogenic differentiation of BM-MSCs by inhibiting Hedgehog signaling pathway.** (**A**) Functional annotation of BM-MSCs lncRNAs at days 0, 7, and 14 of osteogenic differentiation by GO enrichment analysis based on the data obtained from Microarray analysis. (**B**, **C**) Volcano plot of the up-regulated lncRNAs (Red) and down-regulated lncRNAs (Green). (**D**) Protein expression of HHIP, Gli1, Gli2, RUNX2, and osteocalcin in BM-MSCs transfected with HHIP-AS1 or si-HHIP and treated with PF-5274857. (**E**) ALP and (**F**) alizarin red staining after 14 days of osteogenic induction of BM-MSCs. The histogram data for each group are the average of three independent replicates; bars represent standard deviation; ***P < 0.001.

To further explore the signaling pathway downstream of lncRNA HHIP-AS1/HHIP, we analyzed the microarray results in depth. GO enrichment results showed that as osteogenic differentiation progressed, the smoothened signaling pathway (hedgehog signaling pathway) became more active (Figure 6A). Volcanic map showed that HHIP was down-regulated during osteogenic differentiation of BM-MSCs (Figure 6B, 6C). Meanwhile, the key molecules of hedgehog signaling pathways SMO, Gli1 and Gli2 were continuously up-regulated (Figure 6B, 6C). The above analysis implied that HHIP might inhibit osteogenic differentiation by regulating the hedgehog signaling pathway. The results of western blot showed that the expression of HHIP was increased after overexpression of lncRNA HHIP-AS1, and Gli1, Gli2, RUNX2 and osteocalcin were significantly decreased, while these effects caused by lncRNA HHIP-AS1 overexpression was reversed after knockdown of HHIP. PF-5274857 (a potent and selective antagonist that can inhibit the Hedgehog signaling pathway) [26] further abolished the effects conferred by HHIP knockdown (Figure 6D). ALP and alizarin red staining results showed the osteoblastic differentiation of BM-MSCs was suppressed with lncRNA HHIP-AS1 overexpression, but the effect by lncRNA HHIP-AS1 was reversed by HHIP knockdown (Figure 6E, 6F). PF-5274857 conversely abolished the effect caused by HHIP knockdown. These results indicated that lncRNA HHIP-AS1/HHIP may inhibit osteogenic differentiation of BM-MSCs by regulating Hedgehog signaling pathway.

## DISCUSSION

Some studies have reported the functions of lncRNA in osteogenic differentiation of mesenchymal stem cells [27, 28]. It is found that lncRNA HOTAIRM1 functions as a critical regulator to promote osteogenesis of MSCs. The loss of HOTAIRM1 significantly inhibits the calcium deposition and ALP activity of MSCs [14]. Additionally, lncRNA SNHG1 regulates the p38 MAPK pathway through Nedd4, and inhibits the osteogenic differentiation of BM-MSCs [13]. However, the role of lncRNA HHIP-AS1 in osteogenic differentiation of BM-MSCs has not been fully studied. Our study found that lncRNA HHIP-AS1/HHIP inhibits osteogenic differentiation of BM-MSCs by inhibiting the Hedgehog signaling pathway for the first time.

BM-MCSs are progenitor cells of osteoblasts in bone marrow. We performed high-throughput microarray on day 0, 7 and 14 after induction of osteogenic differentiation. The results showed that the expression pattern of lncRNAs and mRNAs were extensively altered during osteogenic differentiation. Whole genome expression analysis has revealed that the differentially expressed genes in MSCs involve in skeletal development and bone formation [29]. Among the differentially expressed lncRNAs, lncRNA HHIP- AS1 had the most significant down-regulation during osteogenic differentiation. A previous study also reported the down-regulation of lncRNA HHIP-AS1 during osteogenic differentiation, but they did not further explore the role of HHIP-AS1 in osteogenic differentiation of BM-MSCs [2]. Apart from lncRNA HHIP-AS1, HHIP also showed dramatic reduction during osteogenic differentiation in our study. Furthermore, we confirmed that the reduction of HHIP was associated with lncRNA HHIP-AS1.

This study revealed an important molecular mechanism by which lncRNA HHIP-AS1 positively regulated the HHIP expression. We found that lncRNA HHIP-AS1 bound to HHIP mRNA through complementary base pairing. This interaction enhanced the stability of HHIP mRNA, whereby increasing the HHIP expression. It is known that some mRNAs are degraded before translation, resulting in the reduced protein levels. It is reported in more studies that the interaction between lncRNA and mRNA enhances the stability mRNA, resulting in higher protein levels. Zhang et al., found that lncRNA DSCAM-AS1 binding to dCTP pyrophosphatase 1 (DCTPP1) mRNA increased the stability and the DCTPP1 protein level. By increasing DCTPP1, lncRNA DSCAM-AS1 plays an important role in the development of breast cancer [30]. Similar molecular mechanism was also reported in the interaction between lncRNA PXN-AS1-L and SAPCD2 [31]. In this study, the overexpression of lncRNA HHIP-AS1 indeed increased the HHIP expression. However, lncRNA HHIP-AS1 knockdown only decreased HHIP protein level in cell membrane, but not in the whole cells. We propose other mechanisms that lncRNA HHIP-AS1 also affects the distribution of HHIP protein in cell fraction.

This study further revealed that lncRNA HHIP-AS1 enhances HHIP mRNA stability by recruiting ELAVL1. ELAVL1, also known as HuR, plays a role in stabilizing mRNA, thus it has been implicated in a variety of biological processes as well as diseases. For example, HuR is increased in human or mouse fibrotic livers. HuR promotes the migration of BMSCs by increasing S1PR3 mRNA stability and expression, which may affect liver fibrosis [32]. With the help of HuR, lncRNA EGFR-AS1 enhances the mRNA stability of epidermal growth factor receptor. As the result, lncRNA EGFR-AS1 promotes cell growth and metastasis in renal cancer [33]. Our study found that knockdown ELAVL1 abolished the effect of lncRNA HHIP-AS1 on enhancing HHIP mRNA stability, suggesting that ELAVL1 is implicated in the regulatory effect of lncRNA HHIP-AS1 on HHIP mRNA stability.

The critical function of Hedgehog signaling in bone formation has been identified in many studies in the past two decades [34–36]. The most important upstream proteins in the Hedgehog signaling pathway include SHH, IHH and DHH. In the early stage of development, SHH regulates the patterning and growth [18, 19]. IHH functions later during endochondral bone formation in limb development. The expression of HHIP is induced by the Hedgehog signaling pathway. When the HH signaling pathway is over-activated, the up-regulation of HHIP inhibits the activity of Hedgehog signaling pathway and results in a negative feedback regulation. HHIP binds with SHH in cell membrane and blocks the activation of the Hedgehog signaling pathway [37]. In the present study, we found that the downstream molecules of Hedgehog signaling pathway, Gli1 and Gli2, were down-regulated after the overexpression of lncRNA HHIP-AS1, which suppressed the osteogenic differentiation of BM-MSCs. However, silencing HHIP blocked the effects of lncRNA HHIP-AS1. Blockage of Hedgehog signaling pathway by PF-5274857 further abolished the effect of HHIP knockdown on lncRNA HHIP-AS1 function in osteogenic differentiation. All these results suggested that lncRNA HHIP-AS1 suppressed osteogenic differentiation of BM-MSCs by up-regulating HHIP to suppress Hedgehog signal.

It is reported that activation of Hedgehog signal suppressed osteogenic differentiation of human multipotent adipose-derived stem cells (hMADS) [38]. It is possible that Hedgehog signal plays different roles in osteogenic differentiation between BM-MSCs and hMADS. Previous study shows that the osteogenic differentiation of hMADS is primarily dependent on the method of PCR [38], but mRNA levels do not always reflect the protein levels. Therefore, the role of Hedgehog signal in osteogenic differentiation of hMADS requires further verification.

**Figure 7:**
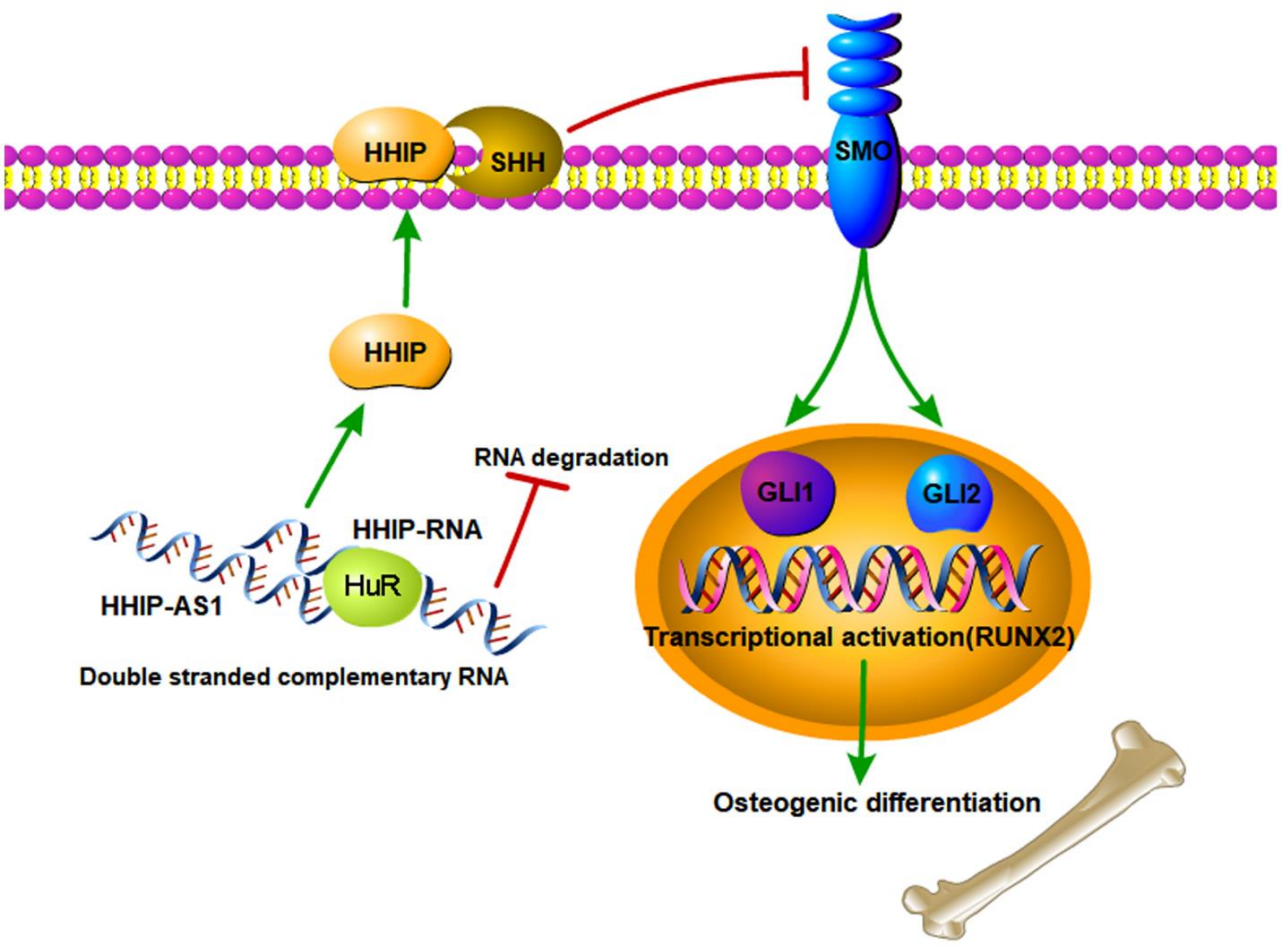
**The schematic diagram of lncRNA HHIP-AS1/HHIP modulating osteogenic differentiation of BM-MSCs.** lncRNA HHIP-AS1 bound to HHIP mRNA through complementary base pairing. This interaction increased ELAVL1 binding to HHIP mRNA and thus improved the mRNA stability and expression. HHIP protein can binds to SHH, which suppresses the activation of SMO by SHH. As the result, osteogenic differentiation of BM-MSCs induced by Hedgehog signal is suppressed.

The shortcoming of this study is that there is no animal experiment to verify the results from the cell study. In the cell study, we revealed that the reduced lncRNA HHIP-AS1 during osteogenic differentiation process plays an important role in osteogenic differentiation of stem cell, because lncRNA HHIP-AS1 stabilized HHIP RNA by ELAVL1 and thus promoted HHIP protein levels, which suppressed osteogenic differentiation of BM-MSCs by inhibiting Hedgehog signal. The schematic diagram is shown in Figure 7. Aging related diseases include osteoporosis, diabetes and degenerative arthritis. Osteoporosis can lead to brittle bones and increase the risk of fracture. Among various treatment methods, stem cell transplantation has attracted much attention. Mesenchymal stem cells have become promising candidates for regenerative medicine to repair non-healing bone defects due to their availability. However, the limited osteogenic differentiation potential has greatly hindered the clinical application of mesenchymal stem cells in bone repair. Our study indicates that intervening the expression of lncRNA HHIP-AS1 and HHIP in mesenchymal stem cells can promote osteogenic differentiation, and provides a possible target and theoretical basis for stem cells to treat the above diseases.

In summary, we investigated the role of lncRNA HHIP-AS1 in osteogenic differentiation. Our findings showed that overexpression of lncRNA HHIP-AS1 increased HHIP protein level by ELAVL1 and then suppressed Hedgehog signal, resulting the inhibition of osteogenic differentiation of BM-MSCs.

## Supplementary Material

Supplementary File 1
