# Supplementary material for: lncRNA HHIP-AS1/HHIP modulates osteogenic differentiation of BM-MSCs by regulating Hedgehog signaling pathway
Source: Aging (Albany NY). 2022 Nov 14;14(21):8839–55. doi: 10.18632/aging.204381 (PMC9699766; doi:10.18632/aging.204381)
Supplement: Supplementary File 1 [file aging-14-204381-s001.doc]

**Supplementary File 1.**

**Homo sapiens hedgehog interacting protein (HHIP), mRNA**

NCBI Reference Sequence: NM_022475.2

[Go to:](https://www.ncbi.nlm.nih.gov/nuccore/NM_022475.2#goto324072750_0)

LOCUS NM_022475 3555 bp mRNA linear PRI 03-JUN-2018

[CDS](https://www.ncbi.nlm.nih.gov/nuccore/NM_022475.2?from=681&to=2783) 681..2783

[exon](https://www.ncbi.nlm.nih.gov/nuccore/NM_022475.2?from=1&to=959) 1..959

ORIGIN

1 **ggccgccggg gcccggggga cgcg**ctcgcg cgggggccgc cccctcccct tccctccacc

61 ctgggcgggg gcgcgcgaga agcggtgacg tcaaggggcg cgctgtggca gcacctcccc

121 gcgcgctagt taaaaagaag aagaaaagag ggaacgaaac atgagaggct gtgtgagaag

181 ctgcagccgc cggcagagga gacctcagca tcatctagag cccagcgctg gccctgcctc

241 cgcctgcccc gccgccgccg tcgccgtttc tgttcctgct actgtcccac ctaaacaact

301 cccgttacac ggacaagtga acatctgtgg ctgtcctctc cttttcttcc tcctcttcca

361 actccttctc ctcctcccac ttcccagccg cagcagaaag cccccaaccc aactgacact

421 ggcacaactg caaacggtgt catccgcaca actttatctc gctcctcggg ctcccctaag

481 gcattggacc catcgccgcg tcttttattt tttgcaaagt tgcatcgctg tacatatttt

541 tgtccccgcc acctccctct gtctctggag tgccctacag ccccgcaaac tcctcctgga

601 gctgcgccct agtgcccctg ctgggcagtg gcgttccccc ccatcctccc gcgcccagcc

661 cctgctgctc tgggcagacg atgctgaaga tgctctcctt taagctgctg ctgctggccg

721 tggctctggg cttctttgaa ggagatgcta agtttgggga aagaaacgaa gggagcggag

781 caaggaggag aaggtgcctg aatgggaacc ccccgaagcg cctgaaaagg agagacagga

841 ggatgatgtc ccagctggag ctgctgagtg ggggagagat gctgtgcggt ggcttctacc

901 ctcggctgtc ctgctgcctg cggagtgaca gcccggggct agggcgcctg gagaataaga

961 tattttctgt taccaacaac acagaatgtg ggaagttact ggaggaaatc aaatgtgcac

1021 tttgctctcc acattctcaa agcctgttcc actcacctga gagagaagtc ttggaaagag

1081 acctagtact tcctctgctc tgcaaagact attgcaaaga attcttttac acttgccgag

1141 gccatattcc aggtttcctt caaacaactg cggatgagtt ttgcttttac tatgcaagaa

1201 aagatggtgg gttgtgcttt ccagattttc caagaaaaca agtcagagga ccagcatcta

1261 actacttgga ccagatggaa gaatatgaca aagtggaaga gatcagcaga aagcacaaac

1321 acaactgctt ctgtattcag gaggttgtga gtgggctgcg gcagcccgtt ggtgccctgc

1381 atagtgggga tggctcgcaa cgtctcttca ttctggaaaa agaaggttat gtgaagatac

1441 ttacccctga aggagaaatt ttcaaggagc cttatttgga cattcacaaa cttgttcaaa

1501 gtggaataaa gggaggagat gaaagaggac tgctaagcct cgcattccat cccaattaca

1561 agaaaaatgg aaagttgtat gtgtcctata ccaccaacca agaacggtgg gctatcgggc

1621 ctcatgacca cattcttagg gttgtggaat acacagtatc cagaaaaaat ccacaccaag

1681 ttgatttgag aacagccaga gtctttcttg aagttgcaga actccacaga aagcatctgg

1741 gaggacaact gctctttggc cctgacggct ttttgtacat cattcttggt gatgggatga

1801 ttacactgga tgatatggaa gaaatggatg ggttaagtga tttcacaggc tcagtgctac

1861 ggctggatgt ggacacagac atgtgcaacg tgccttattc cataccaagg agcaacccac

1921 acttcaacag caccaaccag ccccccgaag tgtttgctca tgggctccac gatccaggca

1981 gatgtgctgt ggatagacat cccactgata taaacatcaa tttaacgata ctgtgttcag

2041 actccaatgg aaaaaacaga tcatcagcca gaattctaca gataataaag gggaaagatt

2101 atgaaagtga gccatcactt ttagaattca agccattcag taatggtcct ttggttggtg

2161 gatttgtata ccggggctgc cagtcagaaa gattgtatgg aagctacgtg tttggagatc

2221 gtaatgggaa tttcctaact ctccagcaaa gtcctgtgac aaagcagtgg caagaaaaac

2281 cactctgtct cggcactagt gggtcctgta gaggctactt ttccggtcac atcttgggat

2341 ttggagaaga tgaactaggt gaagtttaca ttttatcaag cagtaaaagt atgacccaga

2401 ctcacaatgg aaaactctac aaaattgtag atcccaaaag acctttaatg cctgaggaat

2461 gcagagccac ggtacaacct gcacagacac tgacttcaga gtgctccagg ctctgtcgaa

2521 acggctactg cacccccacg ggaaagtgct gctgcagtcc aggctgggag ggggacttct

2581 gcagaactgc aaaatgtgag ccagcatgtc gtcatggagg tgtctgtgtt agaccgaaca

2641 agtgcctctg taaaaaagga tatcttggtc ctcaatgtga acaagtggac agaaacatcc

2701 gcagagtgac cagggcaggt attcttgatc agatcattga catgacatct tacttgctgg

2761 atctaacaag ttacattgta tagtttctgg gactgtttga atattctatt ccaatgggca

2821 tttatttttt atcctgtcat taaaaaaaaa agactgttat cctgctacac actcctgtga

2881 tttcattctc ttttattaat ttaaaaataa tttccagaaa tgtgcagatc ctctgtgtgt

2941 atgtcagcat gtttgttcac atatgcacat acacatactc ataaccccta tatgcgttgt

3001 tgcataacag atgatttttt aaaatatata cttccttatg caaagtaatt tacacagaaa

3061 ttccattgta aattgataat ggatttttta tgttactaga agagattatt tgacttccca

3121 ggaattttct gtctgtaatc actaaagtca actttaatag agttttgaaa cagtactgtg

3181 caatccgatg gatctaatta aaaaaaaggc aatattttta tattaaagta ctatactagg

3241 agagaatgtt tcagaactcc ctgatgaatt tctaagtgag caacttgata taaaattgta

3301 atcttcattt ttgtcagtgt atccagttac agaatgctac acacttacct ttttattggc

3361 tgagaaatct ggttatttca tcttaatctc aagattgttt tcaagtgttt tataattaaa

3421 tcataatagc atattttaaa atcaatcttc ctaaaaggtc tgcttttatt gtatatttta

3481 tttaacaata ggcactgggt ttgtgttaca tatttatata ttttatttta tttttataat

3541 atagacatca cctag

**Homo sapiens HHIP antisense RNA 1 (HHIP-AS1), long non-coding RNA**

NCBI Reference Sequence: NR_037595.1

[Go to:](https://www.ncbi.nlm.nih.gov/nuccore/NR_037595#goto326320060_0)

LOCUS NR_037595 1132 bp RNA linear PRI 24-MAY-2018

[gene](https://www.ncbi.nlm.nih.gov/nuccore/NR_037595.1?from=1&to=1132) 1..1132

[exon](https://www.ncbi.nlm.nih.gov/nuccore/NR_037595.1?from=1&to=313) 1..313

[exon](https://www.ncbi.nlm.nih.gov/nuccore/NR_037595.1?from=314&to=1132) 314..1132

ORIGIN >uc003ijq.2 (HHIP-AS1) length=1132

1 **cgcgtccccc gggccccggc ggcc**tccctg ctgcggcggg gaggggcggc tagccgcgac

61 ggcggcggac ggagggggag ggccgcgagc cttcggaacc agcctcgagg gtgctgaacc

121 ccgcgcgccc gcctcgccgc ggcccccggg tgggggcgtc cacggcccgg gttcccggcg

181 ggcttggggc agggacgcag tccggagatt ccgggactgc ccggctgctg cagcccgcag

241 gctcctctct cctccccgct tcccctccgc actgcctggg ctaaaggcgg ccaagctgag

301 gctccgaaag aaaagggaaa attgcaaccc ttgcctacaa ccagactgac agcataattt

361 cttaggaatc aagaatcaat acagggttat ttctttagat taaaccttaa aagccccctt

421 ggaggctgaa gaagcagagg atagccatgc atacacttga acgtgaaaaa tggtcctctt

481 tcattggaga ggtcttcctc ttcagctaaa ccgacagagt ggtgaaaaga agaagggacc

541 ccttgttgac caaaataatc cagacaaatc aaatcggccc tttccagcaa ggagtggaac

601 agcccaaggg tggctagatc acatcttctg ctcacaccac cactgagcaa ccctctccag

661 gactgccacc tatgcatagg tcatgccctg tgcaagagca cataaagtgg gagctgaagt

721 cctgcccagg ctccattcac caagcagagc ttatgttgtt gtgtgtctgt gtctacccag

781 aggatgtggg tcctttttaa aatgtgcacc aatgcatctt gtatgagctg gcaccagctc

841 ggaccatccc accctcatac cctggtccta tccagggtta tattccctac tttagaagaa

901 ttaattattt ttatgaacgt atttagttta ttgtaaaaaa tgaaatatat gacacaaatt

961 gaaattaatt tcagaggtaa aggtcagaat attaccatgt atgtgaatta tgtttgtatg

1021 tatatgtata tatcaagaga gttatatgaa tatacatttt tgtgtatcag aagtgaggac

1081 aatttaactt tgttaagtgt ttgaaataaa attattttga gttttggaat ca

**1. The proteins that bind to HHIP-AS1 was predicted by using the web (**[**http://rbpdb.ccbr.utoronto.ca//index.php**](http://rbpdb.ccbr.utoronto.ca//index.php)**)**

| **Score** | **Relative Score** | **RBP Name** |
| --- | --- | --- |
| 10.3039431 | 100% | [ZRANB2](http://rbpdb.ccbr.utoronto.ca/proteins.php?PME_sys_operation=PME_op_View&PME_sys_rec=2917) |
| 8.7178165 | 100% | [PABPC1](http://rbpdb.ccbr.utoronto.ca/proteins.php?PME_sys_operation=PME_op_View&PME_sys_rec=1262) |
| 7.3693752 | 100% | [FUS](http://rbpdb.ccbr.utoronto.ca/proteins.php?PME_sys_operation=PME_op_View&PME_sys_rec=1289) |
| 7.08652094 | 100% | [SFRS9](http://rbpdb.ccbr.utoronto.ca/proteins.php?PME_sys_operation=PME_op_View&PME_sys_rec=1358) |
| 7.08652094 | 100% | [SFRS9](http://rbpdb.ccbr.utoronto.ca/proteins.php?PME_sys_operation=PME_op_View&PME_sys_rec=1358) |
| 6.6279899 | 100% | [MBNL1](http://rbpdb.ccbr.utoronto.ca/proteins.php?PME_sys_operation=PME_op_View&PME_sys_rec=1507) |
| 6.4668404 | 100% | [EIF4B](http://rbpdb.ccbr.utoronto.ca/proteins.php?PME_sys_operation=PME_op_View&PME_sys_rec=1251) |
| 6.4668404 | 100% | [EIF4B](http://rbpdb.ccbr.utoronto.ca/proteins.php?PME_sys_operation=PME_op_View&PME_sys_rec=1251) |
| 6.33985 | 100% | [KHSRP](http://rbpdb.ccbr.utoronto.ca/proteins.php?PME_sys_operation=PME_op_View&PME_sys_rec=2661) |
| 6.33985 | 100% | [KHSRP](http://rbpdb.ccbr.utoronto.ca/proteins.php?PME_sys_operation=PME_op_View&PME_sys_rec=2661) |
| 4.84132568 | 100% | [RBM4](http://rbpdb.ccbr.utoronto.ca/proteins.php?PME_sys_operation=PME_op_View&PME_sys_rec=1589) |
| 4.84132568 | 100% | [RBM4](http://rbpdb.ccbr.utoronto.ca/proteins.php?PME_sys_operation=PME_op_View&PME_sys_rec=1589) |
| 4.62028767 | 100% | [SFRS1](http://rbpdb.ccbr.utoronto.ca/proteins.php?PME_sys_operation=PME_op_View&PME_sys_rec=1448) |
| 4.62028767 | 100% | [SFRS1](http://rbpdb.ccbr.utoronto.ca/proteins.php?PME_sys_operation=PME_op_View&PME_sys_rec=1448) |
| 4.40359056 | 100% | [ELAVL1](http://rbpdb.ccbr.utoronto.ca/proteins.php?PME_sys_operation=PME_op_View&PME_sys_rec=1258) |
| 4.40359056 | 100% | [ELAVL1](http://rbpdb.ccbr.utoronto.ca/proteins.php?PME_sys_operation=PME_op_View&PME_sys_rec=1258) |
| 4.40359056 | 100% | [ELAVL1](http://rbpdb.ccbr.utoronto.ca/proteins.php?PME_sys_operation=PME_op_View&PME_sys_rec=1258) |
| 4.40359056 | 100% | [ELAVL1](http://rbpdb.ccbr.utoronto.ca/proteins.php?PME_sys_operation=PME_op_View&PME_sys_rec=1258) |

**ELAVL1: ELAV like RNA binding protein 1 [ Homo sapiens (human) ]**

**Official Full Name:** ELAV like RNA binding protein 1

**Ensembl:** ENSG00000066044 MIM:603466

**Also known as:** HUR; Hua; MelG; ELAV1

**Summary:** The protein encoded by this gene is a member of the ELAVL family of RNA-binding proteins that contain several RNA recognition motifs, and selectively bind AU-rich elements (AREs) found in the 3' untranslated regions of mRNAs. AREs signal degradation of mRNAs as a means to regulate gene expression, thus by binding AREs, the ELAVL family of proteins play a role in stabilizing ARE-containing mRNAs. This gene has been implicated in a variety of biological processes and has been linked to a number of diseases, including cancer. It is highly expressed in many cancers, and could be potentially useful in cancer diagnosis, prognosis, and therapy.

**2. The interaction between HHIP-AS1 and ELAVL1 was initially identified by using the web (**[**http://pridb.gdcb.iastate.edu/RPISeq/#**](http://pridb.gdcb.iastate.edu/RPISeq/)**)**

>sp|Q15717|ELAVL1_HUMAN MSNGYEDHMAEDCRGDIGRTNLIVNYLPQNMTQDELRSLFSSIGEVESAKLIRDKVAGHS

LGYGFVNYVTAKDAERAINTLNGLRLQSKTIKVSYARPSSEVIKDANLYISGLPRTMTQK

DVEDMFSRFGRIINSRVLVDQTTGLSRGVAFIRFDKRSEAEEAITSFNGHKPPGSSEPIT

VKFAANPNQNKNVALLSQLYHSPARRFGGPVHHQAQRFRFSPMGVDHMSGLSGVNVPGNA

SSGWCIFIYNLGQDADEGILWQMFGPFGAVTNVKVIRDFNTNKCKGFGFVTMTNYEEAAM

AIASLNGYRLGDKILQVSFKTNKSHK

## Your submission results

# [RNA-Protein Interaction Prediction (RPISeq)](http://pridb.gdcb.iastate.edu/RPISeq/index.html)

| **Interaction probabilities** |  | |
| --- | --- | --- |
|  |  | |
|  |  | |
| Prediction using RF classifier |  | 0.8 |
| Prediction using SVM classifier |  | 0.8 |

**3. The interaction between HHIP-AS1 and ELAVL1 was further identified by using the web (http://service.tartaglialab.com/page/catrapid_group)**

#### Information about the JOB:

ID: 372520
User label: ELAVL1-HHIP-AS1
[Protein sequence](http://crg-webservice.s3.amazonaws.com/submissions/2021-07/372520/output/protein.fasta)
[Transcript sequence](http://crg-webservice.s3.amazonaws.com/submissions/2021-07/372520/output/rna.fasta)
Generated: 2021-07-01 03:35:01.947956

| # | Protein region | RNA region | Interaction Propensity | Discriminative Power | Normalized Score |
| --- | --- | --- | --- | --- | --- |
| 1 | 251-302 | 8-26 | 23.41 | 61 | 3.88 |
| 2 | 251-302 | 32-83 | 22.98 | 59 | 3.81 |
| 3 | 251-302 | 132-183 | 21.89 | 56 | 3.64 |
| 4 | 251-302 | 107-158 | 20.09 | 54 | 3.35 |
| 5 | 251-302 | 126-177 | 19.57 | 52 | 3.26 |
| 6 | 176-227 | 8-26 | 19.56 | 52 | 3.26 |
| 7 | 176-227 | 8-26 | 19.56 | 52 | 3.26 |
| 8 | 176-227 | 32-83 | 19.16 | 52 | 3.20 |
| 9 | 176-227 | 32-83 | 19.16 | 52 | 3.20 |
